# Genomics of extreme environments: unveiling the secrets of survival

**DOI:** 10.1038/s41598-023-48470-1

**Published:** 2023-12-05

**Authors:** Kian Mau Goh, María-Isabel González-Siso, Rajesh K. Sani

**Affiliations:** 1 Faculty of Science, Universiti Teknologi Malaysia, 81310 Johor, Malaysia; 2 Facultade de Ciencias, CICA—Centro Interdisciplinar de Química e Bioloxía, Universidade da Coruña, 15071 A Coruña, Spain; 3 Department of Chemical and Biological Engineering, South Dakota School of Mines and Technology, Rapid City, SD 57701 USA

**Keywords:** Comparative genomics, Ecological genetics

## Abstract

Life on Earth has displayed remarkable adaptability to the harshest environments, spanning polar regions, scorching deserts, abyssal oceans, lightless caves, noxious lakes, boiling hot springs, and nuclear waste sites. These resilient organisms, known as extremophiles or polyextremophiles, owe their survival due to their unique genetic adaptations. This collection, titled ‘Genomics of Extreme Environments’, comprises several articles published in the esteemed journal Scientific Reports. Each article within this collection investigated genetic signature and adaptation in different extreme environments, including the cold polar region, arid desert, oxygen-deprived Tibetan mountains and others. These studies provide invaluable understanding of how life thrives and evolves under extreme conditions, shedding light on genetic mechanisms and adaptation strategies.

Extremophiles, including polyextremophiles that thrive in multiple extreme conditions, are organisms uniquely equipped to not only survive but thrive in Earth’s most daunting environments, enduring extreme temperatures, intense radiation, high salinity, or extreme pH levels. While much research has focused on extremophilic microorganisms, it is crucial to recognize that animals and insects also possess remarkable adaptations to such hostile surroundings, deserving of further investigation. One noteworthy example is the tiny tardigrade, often referred to as the water bear, which showcases polyextremophilic traits by surviving extreme cold, scorching heat, high atmospheric pressure, radiation exposure, and even the vacuum of space^1^. There are more than a thousand of articles dedicated to tardigrades, highlighting the extensive research conducted on these remarkable organisms. Nevertheless, there is still much more to uncover about extremophilic organisms. Similarly, in the case of penguins, fishes, starfish, flying insects, sheep, and microbes, comprehensive studies emphasize the ample opportunities for further exploration and comprehension of the incredible adaptations exhibited by these diverse life forms in extreme environments.

Three of the articles in this collection are about living organisms in Antarctica, shedding light on their remarkable adaptations to the extreme conditions of this icy continent. The first article focused on gentoo penguins, investigating their genetic diversity and population dynamics with a particular emphasis on their mitochondrial genome^2^. In the second article, titled ‘Novel mitochondrial genome rearrangements including duplications and extensive heteroplasmy could underlie temperature adaptations in Antarctic notothenioid fishes’, Minhas et al. presented interesting findings on mitochondrial genome heteroplasmy in Antarctic notothenioid fish^3^. Utilizing cutting-edge long-read sequencing technologies, the authors assembled and annotated the mitogenomes of five Antarctic notothenioids, revealing the presence of atypical mitogenomes in some species. This study sheds light on the possible role of mitochondrial genome rearrangements and heteroplasmy in the temperature adaptations of these fishes. Moreover, this research team has recently contributed an in-depth analysis of the evolutionary radiation of notothenioid fish, presenting new genome assemblies for 24 species and establishing a time-calibrated phylogeny using comprehensive genome-wide sequence data^4^. Another intriguing inhabitant of Antarctica, *Parochlus steinenii*, the only flying insect native to this frigid region, is the subject of the third article^5^. Through extensive comparative genomic analyses, researchers explore how this insect has adapted to extreme cold temperatures. They identify 68 gene families critical for various biological processes, such as the innate immune system, response to unfolded proteins, protein stability, unsaturated fatty acid metabolism, and DNA packaging. In particular, the expanded acyl-CoA delta-desaturase and Hsc70 gene families exhibited signatures of phylogenetic instability and positive selection after multiple gene duplications, suggesting their possible importance in the adaptation of *Parochlus steinenii* to cold environments. Together, these articles contributed to our understanding of the genetic mechanisms that allow organisms to thrive in extremely cold environments.

Investigating starfish is of paramount importance, as it elucidates their integral role in upholding the equilibrium of marine ecosystems, uncovers their astonishing biodiversity, delves into their regenerative capabilities, and enables their use as vital indicators of environmental health in our oceans^6^. In a study conducted by Sun et al., they presented the recovery of five mitochondrial genomes, highlighting genetic differences between deep-sea and shallow-water sea stars^7^. This study is significant because it provides valuable insights into their phylogenetic relationships and evolutionary history, explaining selection pressure on the mitogenomes of deep-sea starfish and shedding light on the genetic basis of their adaptation. The authors observed a significantly higher A + T content in the mitochondrial DNA of deep-sea starfish compared to their shallow-water counterparts, suggesting genetic distinctions between these two groups.

In the current collection, two articles focus on sheep research. One of these articles explores the adaptation of indigenous sheep breeds in the southern margin of the Taklimakan Desert^8^, an area characterized by low rainfall, heavy sandstorms, sparse vegetation, and a challenging ecological environment. Researchers utilized a combination of transcriptomic and genomic analysis to compare these resilient sheep to normal agro-climatic breeds, examining aspects related to immunity, vision degeneration, and reproduction rates. The study also proposed genetic mechanisms that underlie this adaptation. Additionally, it shed light on the pathways involved in livestock adaptation to extreme desert environments, including ion channel regulation, MAPK signaling, and PI3K-Akt signaling^8^. In a separate study, another group of researchers published an article titled ‘Comparative analysis of long noncoding RNA and mRNA expression provides insights into adaptation to hypoxia in Tibetan sheep’^9^. These researchers collected samples from animals living in high-altitude regions characterized by low oxygen levels and a sheep from low altitude. The challenging environmental conditions in these regions make Tibetan sheep an ideal subject for studying genetic adaptation to high-altitude hypoxia. The researchers aimed to uncover the genetic mechanisms involved in the adaptation process by investigating the expression profiles of long noncoding RNAs (lncRNAs) and messenger RNAs (mRNAs) in the liver and lung tissues. From the comparative transcriptomic study, the authors identified a set of 247 putative functional candidate genes. Additionally, several positively selected genes within or regulating key pathways were identified for energy metabolism. The findings also have implications for further research on genetic resilience and adaptation in other high-altitude animal populations. Together, the findings from both studies on sheep^8,9^ provide valuable molecular markers for adaptability, laying an important foundation for future research and breeding strategies aimed at improving the productivity and resilience of livestock in harsh environments. Furthermore, these studies align with Sustainable Development Goal concepts related to sustainable agriculture, food security, and resilient communities in challenging terrains.

Hyper-arid refers to an extremely arid or extremely dry climate characterized by exceptionally low rainfall. The Atacama Desert, known as one of the driest places on Earth, serves as an example of a hyper-arid region. Numerous researchers have conducted studies related to the diversity, biogeochemical cycles, enzymes, and potential applications of extremophiles and their macromolecules, using samples collected from the extensive desert stretch spanning over 1000 km along northern Chile^10–13^. In an article of this collection, the authors explored the fascinating realm of microbial communities in halite nodules found in Atacama Desert^14^. They conducted a transplant experiment, relocating halite nodules from a control site in the Atacama Desert to three other sites within the same region. The researchers collected environmental data from each site and conducted a comprehensive analysis of taxonomic and functional changes in the microbial communities, including the exploration of metagenome-assembled genomes, after one year. The study revealed that in sites characterized by extreme dryness, frequent wet/dry cycles, and colder conditions, there was a significant increase in the relative abundance of salt-in strategists, predominantly represented by haloarchaea. These opportunistic microorganisms with salt-tolerant strategies belonged exclusively to the archaea domain and showed site-specific distribution, indicating their adaptation to the new environmental conditions following the transplant.

In summary, this collection has provided a captivating exploration into the genetic exploration of organisms thriving in harsh conditions. By focusing on diverse environments such as the cold polar region, deep sea, arid desert, oxygen-deprived Tibetan mountains, these studies have significantly advanced our understanding of how life adapts and survives in extreme circumstances. As we look ahead, this field offers exciting opportunities for future research. These genetic insights may find applications in biotechnology and environmental conservation. With ongoing dedication and interdisciplinary collaboration, genomics in extreme environments promises to unlock new horizons.
